# Older people at the beginning of the COVID-19 pandemic: A scoping review

**DOI:** 10.25646/7857

**Published:** 2021-04-30

**Authors:** Beate Gaertner, Judith Fuchs, Ralph Möhler, Gabriele Meyer, Christa Scheidt-Nave

**Affiliations:** 1 Robert Koch Institute, Berlin Department of Epidemiology and Health Monitoring; 2 Heinrich Heine University Düsseldorf, Institute for Health Services Research and Health Economics; 3 Bielefeld University, School of Public Health, Department of Health Services Research and Nursing Science; 4 Martin Luther University Halle-Wittenberg, Institute for Health and Nursing Science

**Keywords:** COVID-19, PANDEMIC, SARS-COV-2, OLDER PEOPLE, SCOPING REVIEW

## Abstract

This scoping review focuses on evidence gaps regarding the effects on health, social participation and life contexts of older people at the beginning of the COVID-19 pandemic. It is based on a systematic search strategy of the international literature covering a period between December 2019 and June 2020. The review is supplemented by a search of the websites of selected organisations in Germany (cut-off date: 29 June 2020). Search hits were differentiated by types of publication (empirical study, review, discussion paper). The contents were summarised in tabular form according to topic. The publications mainly discussed the high risks of suffering severe courses of COVID-19 faced by older people, specifically those belonging to certain subgroups. In addition, further main topics were the pandemic’s indirect impacts on physical and mental health, physical and cognitive functions and participation in society. Social isolation, loneliness, reduced levels of physical activity and difficulties in maintaining care were discussed as major health risks. Ageism was an issue that was addressed across all of the identified topics. The publications highlighted the need, but also the opportunity, for raising public awareness of the needs of older people in various life contexts. Publications pointed to the urgent need for research into the biological and social causes of older peoples’ high infection risk and how measures could be adapted in a differentiated manner (infection prevention and control measures, social support, medical and nursing care).

## 1. Introduction

People aged 60 years and older and, in particular, men aged 80 years and older or with pre-existing medical conditions are considered the main risk groups for a severe or fatal Coronavirus Disease 2019 (COVID-19); men are generally considered to be at greater risk than women [1–4]. As a vaccine and effective medication to treat COVID-19 were not available when the pandemic broke out, non-pharmaceutical interventions were key to containing the spread of SARS-CoV-2 and preventing the health system from becoming overwhelmed. As early as March 2020, a number of measures to limit physical contact between people were implemented, first in Italy and later across all European countries, including recommendations or regulations instructing those with respiratory symptoms to self-isolate, closure of schools, kindergartens and care facilities, and wide-ranging restrictions on movement and travel, even involving the enforcement of curfews [5]. To protect their elderly populations, Germany and other countries issued recommendations for visits to and contact with residents of long-term care facilities [6]. In addition, the pandemic made it increasingly difficult to maintain regular outpatient medical care as well as nursing care in the community and in long-term care facilities [7–10].

Against this background, it is to be expected that the COVID-19 pandemic has and will have a variety of consequences for the health and well-being of older people. This publication aims to offer a systematic overview of the situation facing older people during the first six months of the COVID-19 pandemic. The conceptual framework for this analysis is a scientific concept for public health monitoring in the population aged 65 and over [11, 12]. Based on the World Health Organization (WHO) Action Plan on Ageing and Health, three central fields of action and related topics were identified through a structured consensus process. These are: personal factors (physical and mental health, physical and cognitive functioning, health behaviour), activities/participation and environmental factors (health care, nursing care, physical and social environment) [11]. This article considers the effects of the COVID-19 pandemic during its initial phase on the three fields of action mentioned above [11–14]. On this basis, we also aim to identify the areas where obvious evidence gaps exist and to highlight issues that will require more in-depth analysis in the future.

## 2. Methodology

To gain a broad overview, a ‘scoping review’ was conducted based on the framework by Arksey and O’Malley [15] including the following steps: searching for and identifying relevant studies, study selection, charting the data, collating, summarising and reporting the results.

### Identification of relevant studies and study selection

**Figure 1:**
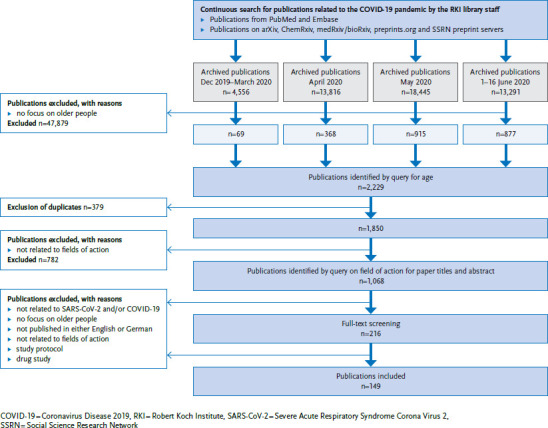
Flow chart for systematic publication selection Source: Own diagram

Since January 2020, the library team at the Robert Koch Institute (RKI) has conducted a continuous search of the international literature on the COVID-19 pandemic in the PubMed and Embase databases. For the database searches, two complexes of search terms were created and linked to identify relevant literature. New terms were continuously added to both complexes as needed (for example, ‘SARS-CoV-2’ was added to the complex of terms related to the virus when the initially nameless virus was named ‘Severe Acute Respiratory Syndrome Coronavirus 2’). Annex Table 1 shows the two complexes. In addition, the team also searched the following preprint servers: arXiv, ChemRxiv, medRxiv/bioRxiv, preprints.org and Social Science Research Network (SSRN). Annex Table 2 shows the search terms used here. All publications identified since 1 December 2019 are archived together with their citations in the reference manager EndNote and have been made available to all RKI staff (Figure 1).

The systematic search for this scoping review was based on publications identified up to 16 June 2020. At this time, four separate literature archives with the identified publications for different time periods were available (Figure 1), which, due to the size of these files, could not initially be transferred to a single database. Therefore, by means of a search in EndNote (keywords ‘aged’ or text words ‘aged’, ‘elder^*^’ or ‘older adult^*^’ in title/abstract), all citations focusing on the target group of older people were identified within the four separate archives. The authors then transferred hits from the four archives into a single literature database in EndNote and removed duplicates using an automatic procedure. In this literature database, separate searches were carried out for the three fields of action (personal factors, activities/social participation and environmental factors) (Annex Table 3). All citations identified in this way were screened independently by two persons per field: first, the title and abstract were checked, followed by the full text of the remaining relevant hits. Disagreements on whether to include a publication were resolved through discussion or by involving other members of the review team. For this two-stage review, the following exclusion criteria had been defined in advance: (1) unrelated to SARS-CoV-2 or COVID-19, (2) no focus on older people, (3) not published in English or German, (4) unrelated to one of the three fields of action mentioned above, (5) study protocol and (6) drug study.

In addition, the websites of preselected German organisations (e.g. professional societies, self-help organisations or research networks) were searched for publications related to the health of older people in connection with COVID-19 released up to and including 29 June 2020. A total of 14 organisations were selected due to their specific connection to the health of older people or to the health effects of the COVID-19 pandemic. Annex Table 4 contains a list of these organisations. Here too, relevant publications were selected independently by two persons taking into account the six predefined exclusion criteria.

### Collating, summarising and reporting the results

The collated and summarised results were presented in tabular form. The results of the literature search in international databases (Table 1) and the results of the internet search on the websites of selected organisations in Germany were analysed separately (Table 2). To increase readability and avoid redundancies with the result table, the summary of the results is presented below without references.

Results from the international systematic literature search were sorted based on topic, methodology and main results. In addition, all included publications were categorised according to publication type (discussion paper, review or empirical study) and the publication’s country of origin or the first author documented.

Since the scoping review aimed to provide a summary of existing scientific knowledge, the next step was to categorise the main content described in the individual publications and assign them to the corresponding topic areas within the fields of action in accordance with the underlying scientific framework concept [11, 12]. For the field of action ‘participation and activities’, for example, nine papers discussed ‘Physical activities in the context of contact and movement restrictions’ and the results of the papers (or the line of argument in discussion papers) all referred to a reduction in physical activity levels following the implementation of social distancing measures. The tabulated summary of the results provides the publications sorted by content category. Likewise, the total number of publications and the proportion of empirical publications are shown by content category, health domain and field of action (Table 1).

Statements retrieved from selected German organisations were listed separately (Table 2) with title, reference and responsible organisation; the central topic was described using key words.

## 3. Results

### Search result

By 16 June 2020, a total of 50,108 publications on SARS-CoV-2 infections or COVID-19 had been archived in four separate literature databases at the RKI (Figure 1). Of these, 47,879 publications were excluded via search query in EndNote as they were not focused on older people. Of the remaining 2,229 publications, 379 duplicates were excluded using an automated procedure. The remaining 1,850 publications formed the basis of the systematic research. The initial search for the fields of action in the literature database using EndNote resulted in a total of 1,068 hits (for all fields of action). The first review (title and abstract) led to the exclusion of 852 publications; the full texts of 216 publications were checked for inclusion.

A total of 149 publications were included for this paper. Of these, 95 were discussion papers [16–110], four reviews [111–114] and 50 empirical studies [115–164]. A total of 77 publications were identified for the field of action ‘personal factors’, 71 for ‘environmental factors’ and 26 for ‘participation and activity’. Some publications were relevant for several fields of action and their results have therefore been included under several fields of action.

The 149 publications came from the following 26 countries: 52 from the USA [16, 18, 24, 30, 31, 39, 42, 49, 51, 52, 56, 57, 59–62, 73, 77–80, 83, 85, 91–93, 95–98, 100, 103, 104, 107–112, 115, 117–119, 123, 135, 143, 144, 149, 152, 157, 160, 162], 13 from China [36, 72, 102, 105, 120, 137–139, 147, 153, 154, 163, 164], 13 from Italy [26, 33, 34, 40, 41, 43, 55, 58, 121, 131, 141, 146, 148], ten from the United Kingdom [21, 29, 44, 48, 63, 89, 113, 130, 136, 161], eight from Canada [17, 20, 45, 47, 54, 66, 94, 156], seven from Spain [67, 74, 90, 128, 129, 140, 150], six from India [22, 23, 35, 99, 145, 155], five from Germany [46, 53, 86, 124, 132], five from Switzerland [32, 68, 76, 81, 82], four from Australia [38, 101, 142, 159], four from Brazil [71, 87, 88, 125], two from Bangladesh [64, 75], two from Belgium [122, 133], two from France [37, 127], two from Ireland [84, 114], two from Israel [70, 158], two from Norway [50, 151], two from Taiwan [65, 106] and one publication each from Chile [69], Ghana [19], Japan [116], the Philippines [28], Thailand [25], Turkey [27], Hungary [134] and Vietnam [126].

The narrative synthesis revealed different topics for the different fields of action, which are listed in Table 1 together with the associated literature. The aim of this scoping review was not providing a detailed analysis of the results.

### Field of action ‘Personal factors’

There were 77 hits for the ‘personal factors’ field of action. These included 37 discussion papers [20, 22, 23, 25, 26, 28, 30–32, 37, 38, 42, 60, 63, 65, 66, 69–71, 73, 81, 82, 84, 86, 87, 89–93, 97–99, 101–103, 105], two reviews [112, 113] and 38 empirical studies [115–122, 124, 125, 127, 129, 131–134, 136–138, 140–142, 145, 147–151, 153–159, 161, 163, 164].

For physical health, some papers described specific subgroups at particularly high risk of suffering a severe or fatal COVID-19 and highlighted that biological rather than chronological age should be used to assess a person’s risk of suffering severe illness as a result of contracting COVID-19. Reviews discussed the genetic, hormonal and immunological factors potentially contributing to the higher risks faced by older people following an infection with SARS-CoV-2. Publications emphasized the need for further research to better understand age-specific changes to the immune system, also regarding the effectiveness of vaccinating older people against COVID-19. Case studies show that older persons may develop atypical symptoms of COVID-19 (i.e. other symptoms may be present). Reduced levels of physical activity, social isolation and loneliness, as well as changes to overall medical care brought about by the COVID-19 pandemic are expected to lead to a deterioration in the physical health of older people. This risk is believed to be particularly great for older people suffering from cancers, Parkinson’s disease, dementia, cardiovascular diseases or osteoporosis. The oral health of older people could also deteriorate. A modelling study concluded that continued physical inactivity in otherwise active, prediabetic older people could lead to an increased incidence of diabetes. An empirical study on emergency neurological care to older people during the COVID-19 pandemic indicated worsened prognoses due to older people delaying seeking medical care.

For the ‘mental health’ domain, some publications expressed the fear that pandemic-related loneliness and social isolation could have serious effects. An increase in the incidence of various psychological disorders and symptoms is expected, especially in patients with pre-existing mental illnesses or dementia, and those living in nursing homes or who are admitted to hospital. Limited access to the health care system as well as intensive media coverage of the pandemic that, at times, features negative images of old age could amplify this. On the other hand, a majority of older people reported good mental health, especially compared to younger adults, in the early stages of the COVID-19 pandemic. However, those older people who suffer more pronounced feelings of loneliness reported more symptoms of anxiety and depression; this was also the case for those with chronic conditions.

In the ‘physical functioning’ domain, it is assumed that physical inactivity due to frequent sitting or lying down as well as loneliness will lead to a deterioration in physical functioning. Frailty is considered a risk factor for more severe and even fatal outcomes of a SARS-CoV-2 infection.

Loneliness, isolation, as well as less support and activation are expected to lead to a deterioration in ‘cognitive functioning’, especially in dementia sufferers, hospitalised patients and those who become infected with SARS-CoV-2. People suffering cognitive impairments often find it difficult to understand and implement the recommended hygiene measures, putting them at an increased risk of contracting SARS-CoV-2 (especially nursing home residents). Based on the available study data, it cannot be determined to what extent dementia increases the COVID-19 mortality risk.

For the ‘health behaviour’ domain, there are discussions about how social distancing reduces physical activity, promoting the loss of muscle and endurance and the increase of frailty. With regard to nutrition, older people were described as having difficulty obtaining sufficient healthy food. With regard to alcohol consumption, there is concern that the pandemic could lead to an increase in abuse. However, compared to younger people, older people were less likely to have increased their alcohol consumption during the pandemic. An ecological study found a negative correlation at the regional level between COVID-19 morbidity rates and flu vaccination take-up among older people in the previous year. Therefore, low COVID-19 rates were found in places where many people had received a flu vaccination and vice versa. It was also found that older people with low education levels were less likely to follow infection prevention measures than those with a higher education; likewise, men were less likely than women to follow these measures.

### Field of action ‘Participation and activity’

A total of 26 publications were assigned to the field of action ‘participation and activity’. Of these, 19 were discussion papers [20, 21, 24, 31, 36, 45, 46, 54, 57, 61, 78, 83, 85, 88, 98, 100, 103, 104, 110], two reviews [111, 114] and five empirical studies [116, 127, 128, 152, 162].

These studies were focused on the health consequences of containment measures related to the COVID-19 pandemic, specifically social distancing and movement restrictions, for older people. The majority of the papers in this field highlighted a potentially increased risk of social isolation and loneliness for older people – both in nursing homes as well as those living in their own homes – as a result of social distancing measures. Against this backdrop, some papers discussed the role of different professional groups (for example, social workers and health professionals) and public health care, as well as the potential benefits of information technologies. In order to avoid the social isolation of older people, two studies analysed programmes providing telephone contact. The results showed a high level of satisfaction with the service. In contrast, a Cochrane Rapid Review found no evidence that video calls with older people can reduce social isolation, loneliness or symptoms of depression, but the number of studies and the reliability of the evidence were low.

Generally, the pandemic was described as a challenge for older people. Some publications dealt with the blanket classification of older people as a risk group and described their increased stigmatisation and even discrimination as possible consequences. In addition to social isolation, other challenges of the pandemic included financial losses, temporary loss of support and increased social inequality. Individual publications also described opportunities, such as the increased use and improved handling of technology, the strengthening of family networks or increased social awareness of the concerns of the elderly.

### Field of action ‘Environmental factors’

A total of 71 hits were found for the ‘environmental factors’ field of action. Of these, 57 were discussion papers [16–19, 27, 29, 31, 33–35, 37, 39–41, 43, 44, 46–53, 55, 56, 58, 59, 61, 62, 64–69, 72–77, 79, 80, 83, 84, 94–96, 98, 99, 103, 105–109], two were reviews [112, 113] and 12 empirical studies [115, 123, 126, 130, 135, 139, 143, 144, 146, 150, 157, 160].

The publications considered the settings in which older people live, receive care and how they have been altered by the pandemic. Several empirical contributions analysed infection outbreaks in nursing homes and assisted living settings. Measures of infection control in health care facilities were discussed and suggestions made for coping with social isolation, boredom, reduced contact and quarantine measures. While publications outlined that, as a group, older people have a greater need for protection and support, it remains important to prevent social isolation and ensure the provision of health care services and social support in nursing homes and home care settings. This would require models for cross-sectoral care and the follow-up care of elderly COVID-19 patients. Some publications analysed the challenges health care systems were facing due to the pandemic regarding specific conditions such as hip fractures.

Health care professionals face major challenges in terms of occupational health and safety and workload, especially when caring for people with mental illness or cognitive impairment. Issues related to limited health care resources (including triage and prioritisation), but also palliative care, stereotyping and ageism are also addressed. Issues of social care and security as well as the impact of laws and regulations on older people are also discussed.

### Contributions by organisations concerning the pandemic and older people in Germany

Until 29 June 2020, contributions related to the pandemic and older people were identified on the websites of 13 of the 14 organisations selected. Of the 57 contributions identified, 47 contributions from a total of ten organisations were included in this review [165–211]. They include statements, comments and recommendations related to (1) infection protection for residents and carers in nursing homes; (2) the equipment provided to nursing homes (e.g. with protective equipment, IT infrastructure); (3) visiting bans and restrictions; (4) the situation facing family caregivers in the home; and (5) the public perception of older people in the context of the COVID-19 pandemic. Table 2 provides an overview of these hits.

The contributions on visiting restrictions addressed the changes that took place during the course of the relaxation of social distancing measures, which started later for nursing home residents compared to other groups. In the context of visiting bans, older people’s risk of social isolation and the potential negative consequences were highlighted. Many contributions criticised the vastly different rules that were applied depending on the federal state and the differences regarding the relaxation of restrictions in nursing homes. The situation for family caregivers was also addressed, for example the burdens caused by a lack of care services such as day care facilities, the difficulties for family caregivers who work and the unique burdens placed on relatives caring for people with dementia. Other topics include the demand for a differentiated picture of older people, for example with regard to the risks of SARS-CoV-2 infection, and the avoidance of ageism.

## 4. Discussion

This scoping review aimed to provide an overview of international and German publications on the direct and indirect effects of the COVID-19 pandemic on the health of older people during the first six months of 2020. The main aim of this evaluation was to identify gaps in the evidence concerning the initial phases of the COVID-19 pandemic and, thus, areas requiring further research. Based on a biopsychosocial understanding of health and guided by the public health framework of the WHO Action Plan on Ageing and Health, the effects of the COVID-19 pandemic on the three fields of action – personal factors, participation/activities and environmental factors – were considered [11, 12, 14].

Only a very small proportion of the extremely high number of publications found for the first six months of the COVID-19 pandemic related specifically to older people’s health. The publication hits reveal serious impacts of the COVID-19 pandemic on the health and well-being of older people in all three fields of action and the associated health domains. However, in the initial stages of the COVID-19 pandemic, discussion paper (e.g. statements, position papers) predominated overall, while literature reviews (4/149) and empirical studies (50/149) accounted for only a third of hits. The distribution of empirical studies among the fields of action was skewed, each accounting for just under 20% in the ‘environmental factors’ and ‘participation/activities’ fields of action compared to 49% in the ‘personal factors’ field of action.

Many of the empirical studies were based on cross-sectional online surveys with small convenience samples without any claim to representativeness [e.g. 117, 119, 140, 149, 158, 159], case series [e.g. 132, 133, 138, 150, 154, 163, 164] or individual case studies [e.g. 151, 161]. These studies have only limited validity since other sampling strategies are needed for older and very old people with health impairments, for example, nursing home residents [212–214]. In addition, there is an urgent need for representative population-based studies of older people living at home in different living contexts (e.g. people living alone in private households with different support and care needs, people with different levels of care dependency, family caregivers) in order to assess the consequences of the pandemic for older people. Here, the evaluations of epidemiological data in Germany collected at the RKI during the course of the pandemic (e.g. GEDA 2019/2020-EHIS, COSMO60+, RKI-Corona-Monitoring, MonAge/Health 65+) can contribute to describing the well-being and health status of older people before and during the pandemic [e.g. 215, 216].

Considering the limited time period that was covered by the search (1 December 2019 to 16 June 2020), including only the initial months of the pandemic, it seems plausible that empirical data are predominantly available from countries that were heavily affected early on in the pandemic, such as China [120, 137–139, 147, 153, 154, 163, 164], Italy [121, 131, 141, 146, 148] and Spain [128, 129, 140, 150]. This is especially true for empirical studies analysing nursing homes. Only two out of the 50 empirical studies came from Germany, and both analysed medical care. A point to take into account is that continuous data collection, which is common in other countries, for example in US nursing homes with the Resident Assessment Instrument (RAI), does not take place in Germany. During the pandemic, it was either not possible to collect data in German nursing homes or only possible under difficult conditions [192]. Mandatory documentation requirements were suspended in nursing homes in Germany. In addition, visits and monitoring by the Health Insurance Medical Service (MDK) and internal supervision were drastically reduced. This also applied to visits by physicians. There was thus a lack of social control and information on the quality of care during the pandemic is limited [193]. The same applies to the frequency with which patients were sedated, measures that deprive patients of their freedom were applied, or challenging behaviour in dementia patients occurred. As of the reporting date, there were no empirical data available on the situation in home care from the perspective of those affected, care staff or family caregivers. Such studies are, however, now also available for Germany. An overview of the available evidence is being compiled by the Competence Network Public Health COVID-19. Empirical data on physical health that were identified in the research period are largely limited to the observation of the high risk for a severe COVID-19 for particularly vulnerable groups of older people, including older people in nursing homes and frail older people who have undergone emergency hospitalisation following a hip fracture. In general, the situations faced by older people in different countries are certainly similar. However, due to the differences in health care systems between countries, not all findings, especially regarding limited health care during the COVID-19 pandemic, are directly applicable to the situation in Germany.

Overall, men seem to run a higher risk than women of suffering a severe COVID-19. Very few empirical studies on the possible indirect effects of the pandemic on the physical health of older people were available during the research period. The results indicate that fears of negative health consequences due to reduced physical activity resulting from social distancing measures, but also due to a delayed access to medical care, could prove true. Initial empirical studies on the impacts of the pandemic on older people’s mental health appear not to confirm the blanket expectation of negative consequences [217, 218]. However, during the initial phases of the COVID-19 pandemic, no data on the potential longer-term consequences were yet available.

During the research period, no empirical data were available on the provision of medical care to older COVID-19 patients, nor for acute medical care or for medical and nursing follow-up care for older people recovering from COVID-19. Similarly, no empirical data were identified for assessing how the pandemic impacted the quality of outpatient and inpatient medical care for multimorbid or frail older people, or for developing quality standards. The extent of the short- and medium-term health consequences of the pandemic for older people also remains unclear. Such consequences would include a deterioration in the physical and mental health, as well as the physical and cognitive functions, of older people in nursing homes and private households. Equally unclear is how cause-specific excess mortality will develop and what changes in mortality will occur by place of death. Regarding the vaccination against COVID-19, more research is needed to determine the effectiveness of the various available vaccines, especially in frail and very old people.

A central question, which is also increasingly being discussed in international publications [e.g. 219], is how to encourage empirical research on the above-mentioned questions, in particular research requiring personal contact. There will also be a need to clarify the options for analysing the consequences of COVID-19 based on the data provided by official statistics, routine data and epidemiological studies. To meet these challenges, ideally, a publicly accessible online repository (i.e. a scientific document server) for the systematic compilation of results produced by empirical studies concerning older people will become available, such as the one set up for long-term care (Example: LTC-COVID).

Numerous publications highlighted the fact that the COVID-19 pandemic had made social tendencies towards age discrimination visible, and this issue was addressed across fields of action and health domains. Blanket risk assessments and medical triage of COVID-19 patients on the basis of chronological age were mentioned, as was the indiscriminate application of measures to restrict contact and mobility. Some contributions also warned against paternalistic attitudes indicating expectations on how older people should behave (e.g. the demand for self-isolation), potentially limiting their freedom of choice [46, 54]. There were calls for a social discourse that recognises the heterogeneity of old people as a group and adapts medical, nursing and social care structures to the largely diverse needs of older people and particularly vulnerable groups (e.g. people in need of care, those who are socially isolated or physically and cognitively impaired). Including new technologies and digital media in a process of adaptation that leads to the creation of appropriate infrastructures and services at the individual and municipal level was seen as a challenge, but also as an opportunity.

This scoping review has its strengths and limitations. The search was conducted for the period from 1 December 2019 to 16 June 2020 in literature archives prepared by the RKI and thus refers exclusively to the initial phase of the COVID-19 pandemic. The systematic search relied on the two most important medical and health science databases, PubMed and Embase, as well as several preprint servers. The search terms were broadly discussed and agreed upon by the authors and included keywords as well as free text terms. To provide findings that reflect the situation in Germany, national contributions via the websites of relevant organisations were included in addition to the literature search. For our study objective, i.e. to provide a systematic overview of the current discourse on the situation of older people during the initial stages of the COVID-19 pandemic, this search strategy seems suitable, but does not claim to be exhaustive. An important limitation of our analysis is that the quality of the studies included was not assessed. However, the aim of the review was to take account of the topics and issues being discussed and not to assess the study results. Furthermore, no quality assessment tools are available to judge the quality of discussion papers, which make up the majority of the included papers.

### Conclusion

The results of this systematic literature review up to June 2020 show that a wide range of direct and indirect effects of the COVID-19 pandemic on the health and well-being of older people can already be expected even during the initial stages of the pandemic. There is an urgent need for empirical research, especially implementation and intervention research, that addresses both infection prevention and control as well as the impact of containment measures on older people in a wide range of life situations throughout the COVID-19 pandemic. Individual (intrinsic) health, functional and social resources and external contextual factors, such as living circumstances, medical and nursing care and social support services, must be taken into account. During the COVID-19 pandemic, it has become clear that continuous and systematic health reporting for the population aged 65 and older is needed; it must also be able to take into account the heterogeneity of this age group. For this purpose, a basis of data needs to be established that can be used in a low-threshold manner and enable timely analyses. The COVID-19 pandemic has shown the danger of a generalising and partly discriminatory approach regarding age. The reality of demographic change requires a differentiated and future-oriented approach to this topic in all areas of society.

## Key statements

- Sociodemographic factors and life contexts influence an older person’s risk of suffering a severe course of COVID-19.
- Social distancing measures likely increase the risk of social isolation, loneliness and physical inactivity among older people, with negative consequences for health and functioning.
- Infection prevention and control measures, social support, medical and nursing care must be adapted to the needs of older people in specific life contexts and their life situation.
- The COVID-19 pandemic highlights the threat of age discrimination and the need to raise societal awareness of the fact that older people form a heterogeneous group.
- There is an urgent need of research on the effects of non-pharmaceutical interventions to protect older people.

## Figures and Tables

**Table 1:** Identified literature by field of action or health domain with classification of document types into specific reference areas: discussion papers [16–110], reviews [111–114] and empirical studies [115–164] Source: Own Table

Field of action or health domain	Content of included publications	Number of publications	Number of empirical studies [source]
**Personal factors**			
Physical health	COVID-19 mortality of older people [93, 137, 138], older men [148, 154], hip fracture patients [150], people in nursing homes [115]	7	6 [115, 137, 138, 148, 150, 154]
	Heightened SARS-CoV-2 infection risk for people who live in care homes [134], who maintain family ties [97], or who do not receive information on current hygiene measures due to sensory limitations [22, 23]	4	1 [134]
	Approaches to explain the high risk older people face when infected with SARS-CoV-2; role played by genetic, hormonal and immunological factors, as well as pre-existing conditions [26, 86, 90, 148, 154]	52	[148, 15]
	Scores to map biological age as a relevant COVID-19 mortality risk factor seem more appropriate than chronological age or sex [26, 113]	2	0
	Symptoms of older people with COVID-19 [82, 113, 151, 157, 163]	5	3 [151, 157, 163]
	Effectiveness of vaccination [86]	1	0
	Medication as a risk factor for a severe COVID-19: antihypertensives [87], potential adverse interaction of long-term drug therapy for chronic diseases with hydroxychloroquine as a candidate for drug therapy to treat COVID-19 [156]	2	1 [156]
	Worsening health behaviour as a risk for a deterioration of health [28], worsening of conditions especially due to physical inactivity [92], increase in the number of new cases of diabetes mellitus [136]	3	1 [136]
	Health issues due to non-use of health care (e.g. follow-up appointments, emergency care) [125, 132] or limited access to health care [92]	3	1 [132]
	Health issues due to social isolation or loneliness [65, 98, 113] or due to persistent feelings of fear and being at risk [92]: chronic stress triggered by this weakens the immune system [22, 65]	4	0
	Changes in the health of people with pre-existing conditions: neurodegenerative diseases [60], cardiovascular diseases [89], osteoporosis [99], stroke [102], Parkinson’s disease [25], cardiometabolic risk factors [90]	6	0
	Patients admitted as inpatients with fractures (broken bones) during the pandemic [131, 133], importance of prophylactic measures to prevent fractures during lockdown [164]	3	3 [131, 133, 164]
	Deteriorated oral health with negative consequences for physical health [69, 73]	2	0
Mental health	Incidence of various mental disorders or symptoms such as depression, anxiety disorders, post-traumatic stress disorders, adjustment disorders, sleep disorders, eating disorders, paranoid disorders and suicide due to loneliness and social isolation [22, 23, 28, 37, 38, 42, 63, 65, 98, 101, 103, 105, 112, 113, 145]	15	1 [145]
	Incidence of somatoform disorders, paranoid disorders, anxiety, distrust in the health system due to media reports (i.e. ‘information overload’) [22, 23, 101, 145, 155]	5	2 [145, 155]
	Groups particularly at risk of mental health deterioration: nursing home residents, hospital patients (e.g. visiting bans), people living alone (e.g. with little social support), people with pre-existing mental health conditions and dementia patients [37, 38, 63, 65, 71, 101, 105, 124, 125, 145, 155]	11	4 [124, 125, 145, 155]
	Underdiagnosis and inadequate treatment of mental (pre-)conditions due to limited health care and discrimination (‘ageism’) and its consequences [22, 23, 37, 38, 70, 71, 101, 105], e.g. worsening of pre-existing mental conditions	8	0
	Difficult implementation or non-compliance with hygiene measures due to mental illness [23] or due to overreactions caused by fear and anxiety [22]	2	0
	Majority report no feelings of depression, anxiety or stress [129, 147, 158, 159]; prevalence figures vary and are difficult to compare, highest rates of 37% for anxiety or depression symptoms according to PHQ-9 or GAD-7 instruments [147]	4	4 [129, 147, 158, 159]
	Lower rates of depression and anxiety symptoms compared to younger adults [118, 129, 149], in people without vs. with chronic pre-existing conditions [129], men vs. women [129]	3	3 [118, 129, 149]
	Higher levels of stress felt than younger people [129] or the general population [70]	2	1 [129]
	Positive correlation between loneliness and psychological symptoms such as anxiety, depression or peritraumatic stress [158]	1	1 [158]
	‘Well-being’ as a factor granting resilience against the negative effects of the pandemic [140]	1	1 [140]
Physical functioning	Deterioration of physical functioning due to physical inactivity (especially in case of hospitalisation or in nursing home residents) [20] and loneliness [65, 113]	3	0
	Association between frailty status and severe COVID-19 [81, 122, 161]; e.g. association with mortality [122, 161]	3	2 [122, 161]
Cognitive functioning	Deterioration of cognitive function (dementia) due to loneliness, isolation, less support and activation [22, 28, 30, 37, 38, 65, 103, 113] or poor mental health [65, 105]	9	0
	Groups at particular risk of cognitive function deterioration: dementia patients, hospital patients (with or without COVID-19) and people after SARS-CoV-2 infection [32, 63, 124]	3	1 [124]
	Cognitive impairment (dementia) causes misinformation and makes implementing hygiene measures difficult (e.g. social isolation) [22, 23, 38, 66, 84, 112], thus increasing the risk of a SARS-CoV-2 infection (especially in nursing homes) [84]	6	0
	Unclear link between dementia and the COVID-19 mortality risk [121, 122]	2	2 [121, 122]
Health behaviour	Pandemic could negatively impact health behaviour (e.g. less physical activity, unhealthy diet, more tobacco and alcohol consumption, unfavourable sleep patterns) [28, 65]	2	0
	Lockdown measures lead to a decrease in physical activity [31, 92, 116, 127]	4	2 [116, 127]
	For dietary habits, gender differences are evident, with women more likely to buy extra food and pay more attention to buying products for a balanced diet compared to men [117]; however, (online) shopping presents challenges, which could potentially lead to changes in eating habits [92, 153]	3	2 [117, 153]
	It is assumed that the consumption of alcohol is increasing [91], yet one study found no such evidence [142]	2	1 [142]
	Vaccination: people vaccinated against flu seem to be less likely to contract COVID-19 [141]	1	1 [141]
	Following recommendations to contain the pandemic: older people make an effort to follow recommendations to avoid infection [119], but there are differences by age and education [117, 120]	3	3 [117, 119, 120]
**Social participation and activities**			
	Social distancing measures lead to a reduction in physical activity [20, 88, 100, 103, 104, 111, 116, 127, 152], e.g. reduced participation in activity programmes [127]	9	3 [116, 127, 152]
	Development of video-based interventions to promote physical activity of older people at home [116]	1	1 [116]
	Increased social isolation and/or loneliness due to restricted contact in nursing homes and in own home [21, 24, 31, 36, 45, 85, 98, 100, 103, 104, 111, 127, 152, 162]	14	3 [127, 152, 162]
	Use of information technologies to reduce social isolation and loneliness or to maintain mental health [36, 57, 83, 104, 110, 111, 128], with so far unclear benefits [114, 152, 162]	9	3 [128, 152, 162]
	Challenges and opportunities of the COVID-19 pandemic for older people [78], e.g. concerns about an exacerbation of social inequality (especially in rural areas) due to an unequal distribution of resources and access to new information technologies [61] or societal awareness of the concerns of older people [78]	2	0
	Increased age discrimination or exclusion of older people [46, 54, 98]	3	0
**Environmental factors**			
Health care	Care of patients with pain [94], with glioblastoma [160], with osteoporotic fractures [99], with hip fractures in hospital (surgical and non-operative, with/without COVID-19) [150], with head and neck tumours [95]; oral health and oral health care [69, 73], psychiatric care in the community [105]	8	2 [150, 160]
	Therapy for patients with breathing difficulties, lung function outcomes. Patient reported outcomes [139]	1	1 [139]
	Criteria-based allocation of treatment (‘triage pathway’) for people with type 2 diabetes presenting foot ulcers [146]	1	1 [146]
	Critical discussion of age as a triage criterion [33, 35]	2	0
	Care concepts under pandemic conditions: geriatric [34, 75, 113], geriatric-oncological [43, 55, 80]	6	0
	Barriers to stopping or reducing medication in a planned process (‘deprescribing’) and overcoming these barriers during the pandemic [47]	1	0
	Challenges with emergency care in hospitals under pandemic conditions [96]	1	0
	Recommendations for action on palliative care in nursing homes and at home [50, 68]	2	0
	Infection control practices in long-term care [16, 72, 106]	3	0
	Transmission and appropriateness of symptom-based screening in nursing homes [115, 130, 135, 143, 144] and assisted living [157]	6	6 [115, 130, 135, 143, 144, 157]
	Specifics of testing strategies in older people [113]	1	0
	Contributions from different disciplines on how to manage the pandemic: geriatric research [83], dementia research [27], exclusion from therapy research [113], demography in the planning of non-pharmaceutical interventions in the pandemic in local and national contexts [44], forensics: post-mortem investigations to clarify unknown conditions of older people [40]	5	0
	Multifactorial interventions to strengthen the social and health system with the aim of ensuring well-being, prevention and access to health care [64]	1	0
	Rationing of limited health care resources: advance care planning, prioritisation, prognostic scoring systems, triage [51, 52, 74]	3	0
	Conditions of the health care system (e.g. number of hospital beds, staffing) play a decisive role in the mortality of COVID-19 patients, in addition to other factors (such as the proportion of > 65-year-olds) [126]	1	1 [126]
	Establishment, implementation and evaluation of post-acute care centres for COVID-19 patients from hospitals, emergency departments, homes and nursing homes [67]	1	0
	Pandemic-related reconfiguration of family medicine [58]	1	0
	Adapted care concepts for older people [39, 76, 77], target group-specific education based on needs [79]	4	0
Nursing care	High vulnerability of nursing home residents and spread of infections in nursing homes [17, 29, 41]	3	0
	Public health activities of various cooperating actors to ensure appropriate care for older people [53]	1	0
	Problems in the care of people with psychiatric disorders or cognitive impairment (dementia): implementation of hygiene measures (work overload), ethics (patients’ rights), occupational safety for staff and others (infection control) [37, 66, 84, 112, 123], e.g. consent to SARS-CoV-2 testing, motor agitation, isolation measures, application of physical restraints and sedation	5	1 [123]
	Task of health care providers: developing methods for identifying and taking action to overcome social isolation and loneliness [103]	1	0
	Implications of the pandemic for various settings in which older people are cared for [107]	1	0
Physical environment	Lack of equipment and preparation for assisted living, lack of infection control, staffing [16]	1	0
	Nursing home setting with particularly high risk, which requires intensive testing and infection control measures, adequate staffing and supervision to prevent neglect and violence as there is no social control by the family [56]	1	0
	Need to optimise communication channels between relatives and nursing homes during the pandemic [59]	1	0
	Vulnerability of older people in rural areas as access to social and health services is more difficult under pandemic conditions [61]	1	0
	Take into account assisted living settings in the planning of strategies to counter the pandemic [109]	1	0
Social environment	Vulnerable social networks and social protection of older people under pandemic conditions [19]	1	0
	Responsibility in social care in the UK in light of the pandemic [48]	1	0
	Senior-friendly service provision in the pandemic in all settings [62] and in the community [18], with a special focus on prevention from becoming a victim [49]	3	0
	Changed social environment and daily life, i.e. less social contact and support, due to social distancing measures [31, 65, 98], incl. approaches to overcoming these challenges [31, 65]	3	0
	Laws and decrees under pandemic conditions with positive effects for older people [108]	1	0
	Stereotypes and disparagement of the value of older people’s lives and proposals against age discrimination (‘ageism’) [46, 98]	2	0

COVID-19 = Coronavirus disease 2019, PHQ-9 = Nine-item Patient Health Questionnaire, GAD-7 = Generalised Anxiety Disorder Scale-7, SARS-CoV-2 = Severe Acute Respiratory Syndrome Corona-Virus 2

**Table 2:** Contributions by national level organisations Source: Own Table

Organisation	Title [source]	Published	Topic
German National Association of Senior Citizens’ Organisations e.V. (BAGSO)	Protecting human lives – strengthening cohesion. BAGSO recommendations regarding the spread of the coronavirus [165]	24.03.2020	Equipment and support for people with recognised care needs in nursing homes and at home
	Coronavirus epidemic in Germany: do not abandon people in care! BAGSO statement on the Day of the Older Generation on 1 April 2020 [169]	30.03.2020	Prevention of and services for older people experiencing social isolation
	End the social isolation of people in nursing homes! BAGSO’s urgent recommendations to politicians [170]	27.04.2020	Relaxation and harmonisation of social distancing restrictions
	Improve support for family caregivers during the coronavirus pandemic! BAGSO’s urgent recommendations to politicians [171]	04.05.2020	Support for family caregivers
	Visits to nursing homes: facilities need clear guidelines and more support. BAGSO issues call to the federal states – for the attention of the Federal Government [166]	25.05.2020	Uniform rules for social distancing restrictions in nursing homes and sufficient provision of protective equipment
	Visits to nursing homes: some federal states urgently need to make improvements. An interim assessment by BAGSO four weeks after the federal-state decision to allow such visits again [168]	03.06.2020	Relaxation and harmonisation of social distancing restrictions
	Ensure basic digital services in old people’s and nursing homes. BAGSO’s five demands [167]	18.06.2020	Better digital equipment for nursing homes
Bundesinteressenvertretung für alte und pflegebetroffene Menschen e. V. (BIVA)	Coronavirus – effects on people with recognised care needs [173]	12.03.2020	Information for relatives on the rules at the beginning of the pandemic
	Visiting restrictions in nursing homes due to the coronavirus crisis. BIVA position paper [172]	26.03.2020	Adequateness of social distancing restrictions for nursing home residents
	Coronavirus: increase the protection for nursing home residents! [175]	31.03.2020	Prevent the spread of COVID-19 infections in nursing homes through better equipment (e.g. protective gear) and regular testing for nursing staff
	Coronavirus crisis and the ‘real everyday practice of care’ in inpatient facilities for the elderly. Guest commentary by BIVA member Claus Völker [174]	03.04.2020	Opinion piece on the situation in nursing homes during the pandemic
	Nursing home residents still at risk – family caregivers are systemically relevant [176]	12.05.2020	Improve access to nursing homes for relatives
	Relaxation of bans on visits to old people’s and nursing homes insufficiently implemented [177]	22.05.2020	Reduction of social distancing restrictions in nursing homes
	Sobering survey result: insufficient options to visit nursing homes and the consequences of social isolation are severe [179]	29.05.2020	Reduction of social distancing restrictions in nursing homes, resume audits by medical services and home supervisors
	Visiting arrangements in retirement homes: making conflicts preventable [178]	12.06.2020	Implementation of the amended social distancing restrictions in nursing homes
German Society for Gerontology (DGG)	Coronavirus: how older people can protect themselves or ‘no kiss for grandma’ [186]	13.03.2020	Prevention of COVID-19 infections among older people
	COVID-19 and the elderly: geriatricians present measures to protect and care for the elderly [187]	20.03.2020	Protective measures and better care for older people
	Care bottleneck: geriatricians call for crisis concept for coronavirus risk group [188]	20.03.2020	Protect caregivers against infection in the context of COVID-19
	Supplementary recommendations for geriatric patients in home care to the ‘Decisions on the allocation of resources in emergency and intensive care medicine in the context of the COVID 19 pandemic’ by the DIVI, DGINA, DGAI, DGIIN, DGP^1^, DGP ^2^, AEM [210]	22.04.2020	Allocation of resources in emergency and intensive care medicine in the context of the COVID-19 pandemic
	After the partial lifting of coronavirus restrictions, geriatricians recommend further protection of the elderly: ‘SARS-CoV-2 has not disappeared yet!’ [189]	08.05.2020	Relaxation of social distancing and application of same rules across Germany
German Society of Gerontology and Geriatrics e.V. (DGGG)	Statement by the DGGG: facilitate psychotherapy via phone for elderly and vulnerable female patients during the coronavirus pandemic [191]	18.03.2020	Access to video appointments for elderly and vulnerable patients with mental illnesses
	Statement by the German Society of Gerontology and Geriatrics on the COVID-19 pandemic [190]	31.03.2020	Targeted measures according to individual risks, not exclusively according to age
	Public communication and reporting on ‘Coronavirus & Age’: recommendations of the German Society of Gerontology and Geriatrics (DGGG), Section III (Social and Behavioural Gerontology) [197]	01.04.2020	Recommendations for public communication on age and COVID-19
	Social hardship of older people in the wake of the COVID-19 pandemic: recommendation for the establishment, support and promotion of local emergency initiatives [199]	07.04.2020	Comprehensive establishment of local emergency aid programmes by municipalities
	Joint statement by the sections for Geriatric Medicine (II), Social and Behavioural Gerontology (III), Social Gerontology and Assistance for the Elderly (IV) of the German Society of Gerontology and Geriatrics (DGGG e.V.): enabling participation and a social life for older people despite the coronavirus pandemic [198]	24.04.2020	Promoting the self-determination, participation and social inclusion of older people
	Joint statement by the Social and Behavioural Gerontology (III) and Social Gerontology and Assistance for the Elderly (IV) Sections of the DGGG: participation and care for people with care needs during the coronavirus crisis and beyond [195]	10.05.2020	Participation and care for people with recognised care needs in the context of COVID-19
German Centre of Gerontology (DZA)	Old people are different, including in the coronavirus crisis [209]	06.04.2020	Providing a differentiated image of age, using non-age discriminating language
	Age discrimination and images of age in the coronavirus crisis [208]	07.04.2020	Promoting the physical activity of older people
	Older people and their use of the internet. Implications for the coronavirus crisis [194]	08.04.2020	Minimising the negative effects of social distancing measures for older people
	Risks of bans on social contact, social support and voluntary work by and for older people [196]	08.04.2020	Improving the digital involvement of older people
	Physical activity of older people during the coronavirus crisis [211]	08.04.2020	Stronger orientation towards concrete risks in restrictions and support services
German Alzheimer’s Association (DAlzG)	Coronavirus: considerations of the unique situation facing family members caring for dementia patients [180]	13.03.2020	Increasing support for family carers
	Coronavirus crisis: regulations for visits to nursing homes [181]	18.03.2020	Information on social distancing in nursing homes
	Coronavirus: the German Alzheimer’s Association calls on politicians to take action [182]	25.03.2020	Support for family carers and people with dementia in their own homes
	Protect dementia patients! German Alzheimer’s Association calls for the easing of visiting bans to nursing homes as early as possible [183]	05.05.2020	Relaxation of visiting bans in nursing homes
	Visits to nursing homes: German Alzheimer’s Association calls for binding regulations for all facilities [185]	12.06.2020	Uniform visiting rules in nursing homes
	Closed day care facilities – are family caregivers funding the coronavirus bailout? [184]	17.06.2020	Support for family caregivers
German Network for Evidence-based Medicine (EbM Network)	COVID-19 pandemic: there is no need to move that fast! No experiments with the elderly and chronically ill population without scientific monitoring [192]	27.03.2020	Systematic documentation and care research in the context of social isolation and visit bans
	Coronavirus in German nursing homes – an evidence-free drama in three acts [193]	28.04.2020	Clinical-epidemiological database on COVID-19 through systematic testing in nursing homes, establishment of a registry
Kuratorium Deutsche Altershilfe e.V. (KDA)	The COVID-19 pandemic and the situation of older people in Germany. A statement by the Kuratorium Deutsche Altershilfe (KDA) [200]	07.04.2020	Maintain and enable protection, participation and self-determination of older people and people in need of care
	Social policy perils and missteps during the coronavirus pandemic. On the affirmative reception of coronavirus in the culture, spirit and soul of ‘Policies for the Elderly’ [205]	14.05.2020	Ensure self-determination and participation for older people despite COVID-19 social distancing measures
Competence Network Public Health COVID-19	Should older employees have to stay away from the workplace? Results of a systematic literature search (rapid scoping review) [206]	24.04.2020	Measures to protect older workers in the workplace
	Social isolation as a mortality risk for older people: results of a systematic literature review (rapid scoping review) supplemented by a qualitative survey [207]	18.05.2020	Consequences of social isolation and loneliness for health in nursing homes and the lifting of social distancing measures
Pflegeethik Initiative	Not allowed to live, not allowed to die [203]	26.03.2020	Proportionality of social distancing measures, positions taken by different professions on the topic of dying
	General bans on visits to homes are inhumane!!!! [204]	17.03.2020	General bans on visits to nursing homes and their effects
	Wrong priorities set and ethical principles violated [202]	15.04.2020	Social distancing as compulsory care measures and the consequences for older people
	Urgent coronavirus statement: visiting bans in nursing homes are inhumane and disproportionate. Lift them immediately! [201]	03.05.2020	Social distancing measures are not proportionate, demand for immediate removal

BAGSO = German National Association of Senior Citizens’ Organisations e.V., BIVA = Bundesinteressenvertretung für alte und pflegebetroffene Menschen e.V., DAlzG = German Alzheimer’s Association e.V., DGG = German Society for Gerontology e.V, DGGG = German Society of Gerontology and Geriatrics e.V., DZA = German Centre of Gerontology, EbM-Netzwerk = German Network for Evidence-Based Medicine e.V., KDA = Kuratorium Deutsche Altershilfe e.V., Pflegeethik Initiative = Pflegeethik Initiative Deutschland e.V., DIVI = Deutsche Interdisziplinäre Vereinigung für Intensiv- und Notfallmedizin e.V., DGINA = Deutsche Gesellschaft Interdisziplinäre Notfall- und Akutmedizin e.V., DGAI = Deutsche Gesellschaft für Anästhesiologie und Intensivmedizin e.V., DGIIN = German Society for Internal Intensive Care and Emergency Medicine e.V., DGP^1^ = German Society for Pneumology and Respiratory Medicine e.V., DGP ^2^ = German Society for Palliative Medicine e.V., AEM = Academy for Ethics in Medicine e.V., COVID-19 = coronavirus disease 2019, SARS-CoV-2 = Severe Acute Respiratory Syndrome Corona-Virus 2

## Appendix

**Annex Table 1:** Literature databases and search strings used by library staff at the Robert Koch Institute to identify publications on SARS-CoV-2 and COVID-19 (as of 16 June 2020) Source: Own Table

PubMed
► Search string 1: (“Severe Acute Respiratory Syndrome Coronavirus 2”[Supplementary Con-cept] OR “COVID-19” [Supplementary Concept] OR “spike glycoprotein, COVID-19 virus” [Supplementary Concept] OR “Severe Acute Respiratory Syndrome Coronavirus 2”[tiab] OR ncov*[tiab] OR COVID*[tiab] OR sars-cov-2[tiab] OR “sars cov 2”[tiab] OR “SARS Corona-virus 2”[tiab] OR “Severe Acute Respiratory Syndrome CoV 2”[tiab] OR “Wuhan corona-virus”[tiab] OR “Wuhan seafood market pneumonia virus”[tiab] OR “SARS2”[tiab] OR “2019-nCoV”[tiab] OR “2019 novel coronavirus*”[tiab] OR “2019 novel human coronavirus*”[tiab] OR “coronavirus disease-19”[tiab] OR “corona virus disease-19” [tiab] OR “coronavirus dis-ease 2019”[tiab] OR “corona virus disease 2019”[tiab] OR “2019 coronavirus disease”[tiab] OR “2019 corona virus disease”[tiab] OR “new coronavirus*”[tiab] OR “coronavirus out-break” [tiab] OR “coronavirus epidemic”[tiab] OR “coronavirus pandemic”[tiab] OR “pan-demic of coronavirus”[tiab]) AND (“2019/12/01”[PDAT] : “2099/12/31”[PDAT]) ► Search string 2: (“wuhan”[tiab] or china[tiab] or hubei [tiab]) AND (“Severe Acute Respiratory Syndrome Coronavirus 2”[Supplementary Concept] OR “COVID-19” [Supplementary Con-cept] OR “coronavirus*”[tiab] OR “corona virus*”[tiab] OR ncov[tiab] OR COVID*[tiab] OR sarscov2[tiab] OR “sars cov 2”[tiab])
Embase
► Search string 1: (‘severe acute respiratory syndrome coronavirus 2’:ti,ab OR ‘severe acute res-piratory syndrome coronavirus 2’/exp OR ‘COVID-19’:ti,ab OR ‘COVID 19’/exp OR ‘spike gly-coprotein, COVID-19 virus’:ti,ab OR ncov*:ti,ab OR COVID*:ti,ab OR ‘sars cov 2’:ti,ab OR ‘sars coronavirus 2’:ti,ab OR ‘sars corona virus 2’/exp OR ‘severe acute respiratory syndrome cov 2’:ti,ab OR ‘wuhan coronavirus’:ti,ab OR ‘wuhan seafood market pneumonia virus’:ti,ab OR sars2:ti,ab OR ‘2019 ncov’:ti,ab OR ‘2019 novel coronavirus*’:ti,ab OR ‘novel coronavirus 2019’/exp OR ‘2019 novel human coronavirus*’:ti,ab OR ‘coronavirus disease-19’:ti,ab OR ‘corona virus disease-19’:ti,ab OR ‘coronavirus disease 2019’:ti,ab OR ‘coronavirus disease 2019’/exp OR ‘corona virus disease 2019’:ti,ab OR ‘2019 coronavirus disease’:ti,ab OR ‘2019 corona virus disease’:ti,ab OR ‘new coronavirus*’:ti,ab OR ‘coronavirus outbreak’:ti,ab OR ‘coronavirus epidemic’:ti,ab OR ‘coronavirus pandemic’:ti,ab OR ‘pandemic of coronavirus’:ti,ab) AND 2020:py ► Search string 2: (wuhan:ti,ab OR china:ti,ab OR hubei:ti,ab) AND (‘severe acute respiratory syndrome coronavirus 2’:ti,ab OR ‘severe acute respiratory syndrome coronavirus 2’/exp OR ‘COVID-19’:ti,ab OR ‘COVID 19’/exp OR coronavirus*:ti,ab OR ‘corona virus*’:ti,ab OR ncov:ti,ab OR COVID*:ti,ab OR sarscov-2:ti,ab OR ‘sars cov 2’:ti,ab OR ‘sars coronavirus 2’/exp)

**Annex Table 2:** Preprint servers and the respective search strings used by the library staff of the Robert Koch Institute to identify publications on SARS-CoV-2 and COVID-19 (as of 16 June 2020) Source: Own Table

**Preprint-Server:**
► arXiv: https://arxiv.org/search/advanced?advanced=&terms-0-operator=AND&terms-0-term=COVID-19&terms-0-field=title&terms-1-operator=OR&terms-1-term=SARS-CoV-2&terms-1-field=abstract&terms-3-operator=OR&terms-3-term=COVID-19&terms-3-field=abstract&terms-4-operator=OR&terms-4-term=SARS-CoV-2&terms-4-field=title&terms-5-operator=OR&terms-5-term=coronavirus&terms-5-field=title&terms-6-operator=OR&terms-6-term=coronavirus&terms-6-field=abstract&classification-physics_archives=all&classification-include_cross_list=include&date-filter_by=all_dates&date-year=&date-from_date=&date-to_date=&-date-date_type=submitted_date&abstracts=show&-size=200&order=-announced_date_first&-source=home-COVID-19 ► ChemRxiv: https://chemrxiv.org/search?q=COVID%20OR%20sars-cov-2&sortBy=first_online_date&sort-Type=desc ► medRxiv/bioRxiv: https://connect.medrxiv.org/relate/content/181 ► Preprints.org: https://www.preprints.org/search?-search1=COVID&field1=title_keywords&clause=OR&-search2=sars-cov-2&field2=title_keywords&search_subject_area=&search_subject_sub_area=&date_from=&-date_to=&search_btn ► SSRN: https://www.ssrn.com/index.cfm/en/coronavirus

**Annex Table 3:** Search strings used per field of action Source: Own Table

**‘Environmental factors’ field of action:** (“Social Environment”[MeSH] OR “Social Support”[Mesh] OR “social environment” [TIAB] OR environment^*^[TIAB] OR “social support”[TIAB] OR “health care”[TIAB] OR “Health Services”[MeSH] OR “health service^*^”[TIAB] OR “Nursing Care”[MeSH] OR “nursing care”[TIAB] OR “Residence Characteristics”[MeSH])
**‘Participation’ field of action:** (“social participation”[MeSH] OR “Social Isolation”[MeSH] OR “Leisure Ac-tivities”[MAJR] OR participation[TIAB] OR participat^*^[TIAB] OR “Activities of Daily Living”[MeSH] OR activit^*^[TIAB] OR inclusion[TIAB] OR “Health Literacy” [MeSH] OR “health literacy”[TIAB] OR “Health Behavior” [MeSH] OR “Health Behavior”[TIAB] OR “Health Behaviour” [TIAB] OR “Aging in Place”[TIAB])
**‘Personal factors’ field of action:** (health[MeSH] OR health^*^[TIAB] OR “life expectancy”[MeSH] OR life expectancy[TIAB] OR mortality[MeSH] OR mortality [TIAB] OR “mental health”[MeSH] OR “mental health”[TIAB] OR depression[MeSH] OR depress^*^[TIAB] OR anxiety[MeSH] OR anx^*^[TIAB] OR suicide[MeSH] OR suicid^*^[TIAB] OR frailty [MeSH] OR frail^*^[TIAB] OR “physical function^*^”[TIAB] OR functioning[TIAB] OR cognition[MeSH] OR cognit^*^[TIAB] OR memory[MeSH] OR memory[TIAB] OR dementia [MeSH] OR dementia[TIAB] OR “health behav-ior”[MeSH] OR “health behav^*^”[TIAB] OR “health-related behav^*^”[TIAB] OR lifestyle[TIAB] OR “alcohol drinking”[MeSH] OR alcohol [TIAB] OR smoking[MeSH] OR smok^*^[TIAB] OR exercise [MeSH] OR exercise[TIAB] OR “diet, healthy”[MeSH] OR “healthy diet^*^”[TIAB]) OR sleep[MeSH] OR sleep[TIAB])

**Annex Table 4:** List of selected German organisations Source: Own Table

Federal Association of Senior Citizens’ Organisations e.V. (www.bagso.de) Bundesinteressenvertretung für alte und pflegebetroffene Menschen e.V. (www.biva.de) German Alzheimer’s Association e.V. Self-Help Dementia (www.deutsche-alzheimer.de) German Society of Geriatrics e.V. (www.dggeriatrie.de) German Society of Gerontology and Geriatrics e.V. (www.dggg-online.de) German Society for Nursing Science e.V. (www.dg-pflegewissenschaft.de) German Society for General and Family Medicine e.V. (www.degam.de) German Society for Infectiology e.V. (www.dgi-net.de) German Society for Palliative Medicine e.V. (www.dgpalliativmedizin.de) German Network for Evidence-Based Medicine e.V. (www.ebm-netzwerk.de) German Centre of Gerontology (www.dza.de/informationsdienste.html) Competence Network Public Health COVID-19 (www.public-health-COVID19.de) Kuratorium Deutsche Altershilfe e.V. (www.kda.de) Pflegeethik Initiative Deutschland e.V. (www.pflegeethik-initiative.de)
